# Template-Based Generator for Single-Choice Questions

**DOI:** 10.1007/s10758-023-09659-5

**Published:** 2023-06-07

**Authors:** Nico Willert, Jonathan Thiemann

**Affiliations:** Faculty of Economics and Management Science, University of Leipzig, Leipzig, Saxony Germany

**Keywords:** Question generation, Assessment, Multiple-choice, Generative-software-development, Feature-oriented-engineering

## Abstract

Manual composition of tasks and exams is a challenging and time-consuming task. Especially when exams are taken remotely without the personal monitoring by examiners, most exams can easily lose their integrity with the use of previously done exercises or student communication. This research introduces an approach that incorporates the principles of the generative software development and aspects of the feature-oriented product line engineering process into the field of question creation and generation. The resulting generator can be used to generate single-choice-question-families by means of written question templates. The generated questions within a question-family differ based on the set features and parameters and can be imported into the target learning management system ILIAS. Without much effort, examiners and educators can use the generator to create variants of their questions and deliver them to their students.

## Introduction

Constructing exam questions is without saying a very time-consuming and challenging task for all examiners. Especially when teaching, learning and assessment concepts are moving more towards hand-on approaches with many exercises. This in turn drives educators to use automated and rapid feedback mechanisms further facilitated through smaller scaled learning experiences. In such a way, content is clustered to be absorbed through small units of learning and to be made available on demand, so that learning is neither location- nor time-bound.

Additionally, through the pandemic years, many changes had to be made and new situations had to be accounted for. The COVID-19 pandemic situation not only forced the teaching to be in digital spaces, but also many exams had to be made available in some remote way. This not only meant that in comparison with in person examinations, students were often able to use all of the lecture materials and notes, but this also opened up the possibility for students to easier communicate with each other.

In the specific case of the University of Leipzig, the institution interpreted both German and EU law in such a way that virtually all exams were conducted online. Due to data privacy, no identity check was carried out apart from the login to the exam. Furthermore, it was not allowed that the examinees and their surroundings were monitored. Therefore, examiners had to assume that the students would use aids or solve the exams together. To prevent such actions, many educators started to create multiple versions of the same questions to at least hinder communication. All in all, this requires a lot of time and effort to ensure that the questions possess an equal quality and difficulty. In order to ensure examination fairness, task variations or task specification can be used so that the tasks differ from each other and are designed differently.

The prototype presented in this paper focuses on multiple-choice, or more specifically single-choice questions. In the approach presented here, which is part of an ongoing research, different impulses from different fields are combined. At the forefront of this research stands the paradigm of generative software development according to the principles of Czarnecki (2004), whose aspects are applied to create a generator for single-choice questions. To further approach the creation and generation of examination tasks, feature oriented product line engineering based on Apel et al. (2013) is used to guide the overall development process. Additionally, certain characteristics from domain specific languages are used to model specific use cases for single-choice questions. The main platform for which the prototype was developed is the web-based learning management system ILIAS, which we especially use for e-assessments.

Product line engineering is an approach to software development that emphasizes the reuse of existing assets and knowledge in order to develop and maintain a product line of related software products more efficiently (Apel et al., 2013). In product line engineering, software engineers create a set of core assets that can be used, reused and adapted across multiple products within a product line, rather than starting from scratch for each new product. This approach can save time and resources while also improving the consistency and quality of the resulting products. The core assets in a product line engineering approach typically include requirements, design models, code, and test cases. This is accomplished by identifying and capturing commonalities and variabilities among the products, and then using this information to create a framework that allows for easy customization.

Through the use of certain facets from product line engineering, the proposed approach reuses domain specific knowledge to create a common template with specific fields for each feature that can occur in a question. In order to write these question templates and to integrate the specific knowledge, a domain specific language was developed, which allows to abstract from the technical solution side and to describe the necessary components by using terms of the domain.

A question-template incorporates sections for the name of the question, the author, date, version, as well as other sections for meta-information and sections for the integration of images or code examples. In addition, aspects of the questions can be altered, for example through boundary conditions and definable parameters that personalize the question with the allowable values. In a task section the question is specified and finally the correct and incorrect answers are listed in the true and false sections.

This allows educators to create question-templates from which families or sets of similar questions can be generated. In this way each question is a concrete instance of a template with concrete implementation of common and variable features of the question-family. Based on the defined parameters and boundaries, examiners and educators can control the difficulty, quality and similarity of the tasks.

### State-of-the-Art

The COVID 19 pandemic has brought many challenges to educational institutions, with online and e-learning infrastructure being one of the biggest issues. As a result, there has been a growing interest in incorporating information and communication technologies, including the use of virtual reality technology to provide a more engaging and effective remote learning experience (Sood & Rawat, 2022), and the use of artificial intelligence and data driven approaches. Especially in the specific case of generating questions many different techniques are used (Kurdi et al., 2020; Sewunetie & Kovács, 2022). Creating generators for questions is in and of itself not a new idea. Abd Rahim et al. (2017) references three forms of algorithms to create questions and corresponding exams, namely randomized generation, backtracking algorithms and artificial intelligence. In addition to these, some researchers created tools for the declaration of questions with templates. In this way, the questions and their contents are declared separately and later put together to generate multiple questions based on different data entries (Cruz et al., 2012; Žitko et al., 2009). Besides the template-based generation, other techniques include syntax-based, semantics-based, rule-based, neural-network-based, ontology-based, and reinforcement learning-based question generation (Kurdi et al., 2020; Sewunetie & Kovács, 2022). Most techniques and resulting generators rely on training data due to their strong connection to artificial intelligence and neural networks. Therefore, there are some application areas where the use of these techniques is not feasible because the required amount of data is either not available or would have to be created first.

### Research Gap

The lack of a comprehensive method that can be applied in various fields and educational contexts, independent of particular technologies, is what constitutes the research gap in this area. While there are many state-of-the-art approaches available, they often rely on highly specialized technology or are limited to specific fields. This creates a significant challenge for educators who may need to use a variety of technologies in their teaching or who work in interdisciplinary fields. As a result, there is a need for a more adaptable and flexible approach that can be used in different contexts and with a variety of technologies. Closing this research gap would provide educators with a more accessible and user-friendly approach to task generation that could be used across a range of disciplines and educational settings.

### Research Objectives

The purpose of this research is to develop a tool that supports the creation of exam questions by reusing common traits and parameterization of question aspects. In doing so, we are able to cut down the time and effort necessary to instantiate multiple questions. In addition, it will be shown that the aspects of product line engineering cannot only be used in the engineering context but also transferred to other application fields. Overall, this research aims to contribute to a more efficient, effective, and adaptable approach to exam question creation that can be applied across different fields and disciplines.

### Related Work

Various studies address the generation of exercises in their own way, therefore similar approaches with template-based generators will be discussed. Additionally, we would like to briefly highlight another promising area of approaches with artificial intelligence.

Similar to our approach, Žitko et al. (2009) proposed a template-driven approach for generating questions that is based on previously formalized domain knowledge. The created system separates domain knowledge and question structure in two different templates, so that experts for the domain knowledge create one, and experts for formulating questions can create the other template. This way, it is possible to have the workload divided between examiners, but this also carries the risk of creating two templates that might be difficult to understand and bring together for an external person. The advantage of this approach is, that based on the chosen domain/data and question structure/scheme, the quizzes can be dynamically generated and controlled to include easier or harder questions.

Additionally Cruz et al. (2012) generate math questions, by creating templates written in LaTeX and python. These templates are used to create questions for mathematical formulas, by either randomizing certain aspects or using predefined parameters by connecting the templates to a database. Their software parses the designated text files and defined parameters and replaces the parameters with numeric values or formulas. In this way the package enables authors to easily add, change and delete parameterized exercise templates.

The last publication to mention in the context of templates is Nagasaka (2020). Again, in the mathematical domain, a generator for multiple-choice questions and fill-in-blank questions was created with Katex for Moodle and ported to Jupyter Notebook. With this system, educators are able to parameterize questions with mathematical formulas, so that the system calculates the parameters and computes different answer possibilities. Nagasaka (2020) especially remarks that parameterized multiple-choice are well suited for situations, when quantity of questions is important. Therefore, such questions can be used for self-assessment scenarios, where students can repeat quizzes that consist of similar questions.

Even through these approaches all solve specific problems, the creation of questions is always linked to a specific language like TeX for LaTeX and KaTeX in Cruz et al. (2012) and Nagasaka (2020) or OWL in Žitko et al. (2009). This can make it relatively difficult to create question templates for educators and examiners, who are not familiar with these technologies.

Besides these publications, we want to mention approaches from the field of artificial intelligence namely natural language processing (NLP). Aldabe et al. (2006), Vimalaksha et al. (2021) and Vachev et al. (2022) have created question or distractor generators. Aldabe et al. (2006) processes a corpus of sentences with different NLP-tools. By extracting important information, they create questions or sentences for multiple-choice-questions, fill-in-the-blank or error correction exercises. On the other hand (Vimalaksha et al., 2021) specializes on creating distractors from commented source code. The correct answer is thereby determined from processing the source code and its comments for certain named entities. Based on this and similarly tagged data in the database, distractors are derived. Both these publications show an effective use of NLP for creating questions, but also report the most prominent shortcoming, that these questions tend to have a low quality and problems with the wording of questions and answers. Finally, Vachev et al. (2022) propose a generator for multiple choice question generation called Leaf, based on the use of factual texts. They employ text processing engines that were trained with different datasets. Their generator creates question–answer pairs based on an available text. The generator creates distractors in the second stage of processing, which are combined with the question–answer pairs. Due to the lack of datasets specifically designed for question generation, their generator can currently only produce primarily factual questions.

## Methodology

Described in this paper, the FeatureIDE^1^ plugin for Eclipse was used to create the feature models. The generator itself was developed in C++ by evolutionary prototyping. This way, the general logic for the parser and the domain specific language was implemented first. Then the necessary functionality to compose the parameters was added, followed by the creation of the export facility for the target system. The whole process was guided by the adaption of the process for feature-oriented software product lines by Apel et al. (2013), which is described in the following sections.

### Process Adaption for Question Generation

As described in the introduction, the methodology is based on the paradigm of generative software development and aims to transfer its essential aspects into the domain of question generation. According to Czarnecki (2004), generative software development is an approach to create a system family, which in turn focuses on “a given system that can be automatically generated from a specification” which is usually given in a domain-specific language. Therefore, the question generator shall strive towards the ability to generate similar questions as part of a question family, that get created based on a specification. This relates to a kind of family-based specification, in which specifications are defined, so that they consist of all features, that subsets of products have in common (Thüm et al., 2014). Additionally, Czarnecki states that, “System family engineering distinguishes between at least two kinds of development processes: domain engineering and application engineering”. These processes have been taken up and further developed by other publications. In the following our approach follows and adapts the process of feature-oriented software product lines by Apel et al. (2013).

Apel et al. (2013, p. 19) states “Features, feature selection, feature constraints, and products arise in all kinds of product lines, and are not limited to software product lines.”. It is therefore justified to apply this process to question generation as a form of product line.

For this reason, it is important to clarify the main aspects and process steps that are relevant for the adaption. Firstly, a feature is a characteristic or end-user-visible behavior, that is used to specify commonalities and differences for communication between stakeholders, system structure, reuse and for the software life cycle (Apel et al., 2013, p. 18). Secondly a domain is an area of knowledge that is scoped towards the satisfaction of requirements, which includes a set of concepts and terminology to describe that area and knowledge of how to build software systems in that area (Czarnecki & Eisenecker, 1999).

As Apel et al. (2013) and Czarnecki (2004) we differentiate between domain engineering and application engineering as well as problem- and solution-space, as shown in Fig. 1 from Apel et al. (2013, p. 20). Domain engineering, as the process of analyzing the domain of the product line and development artifacts, is used to define the general framework in which a question and its features are defined. Application engineering, with the goal of developing a specific product for a particular customer’s needs, represent the different questions that are to be engineered for the generator. The problem space, which usually takes the perspective of stakeholders and their problems, requirements and domains, represents the need for creating multiple choice questions for programming education, as well as the e-learning platform that is used. Solution space, covers the design and implementation from the view of developers, which represents on the one hand the resulting generator in the domain engineering and the question-templates for the application engineering. This follows the statement of Apel et al. (2013), that domain engineering is performed once and application engineering is used for every individual product. In tandem with these processes the aspect of quality assurance can either be moved into the domain engineering, or be applied to the entire process. By applying it to domain engineering, this indicates that the generator shall already only be able to create valid questions without the possibility to create invalid questions. When applying to the overall process, each process should enable some sort of possibility to ensure the quality of the generator itself, the questions generated or the question configurations.

**Fig. 1:**
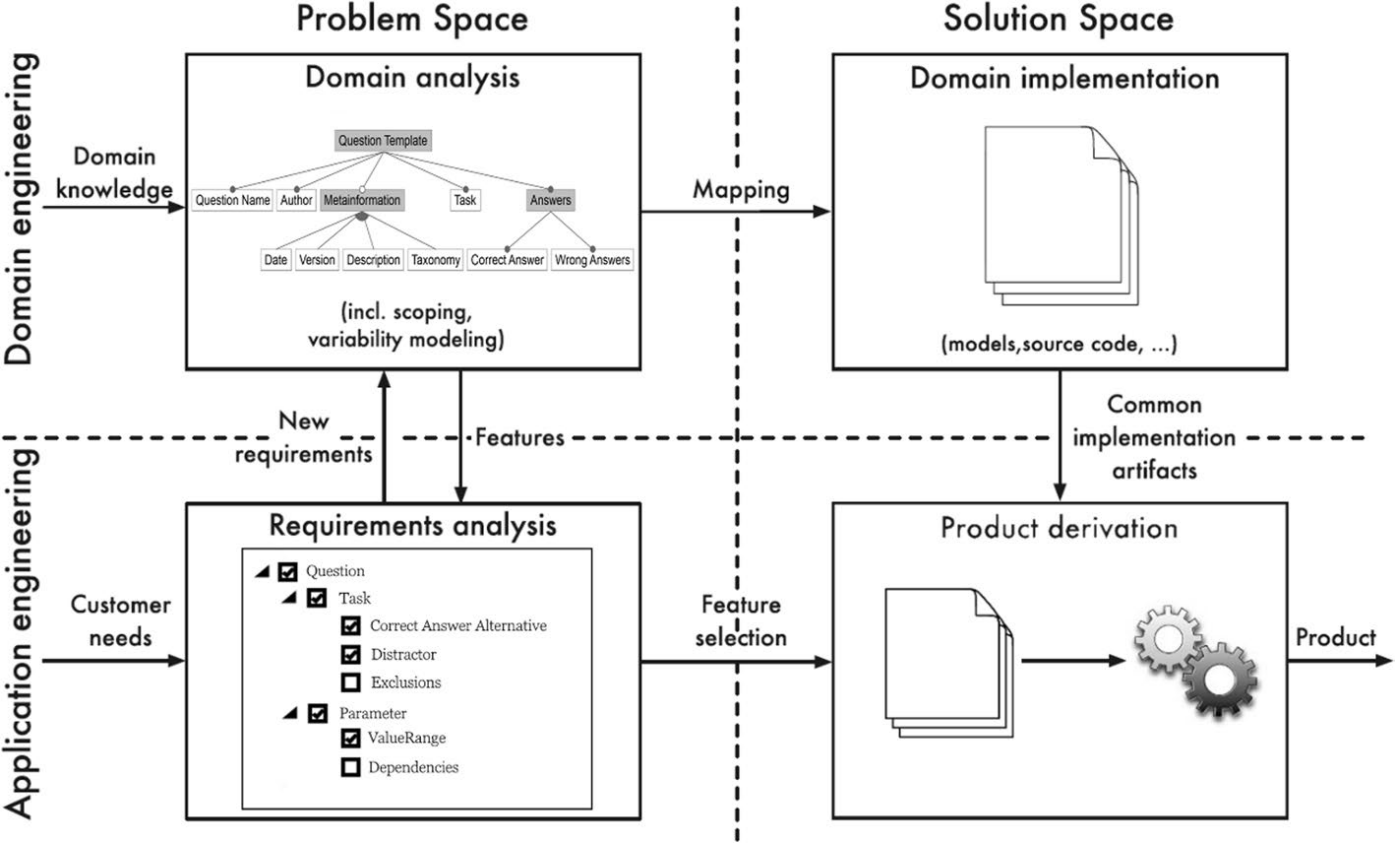
Overview over the engineering process for software product lines, adapted from (Apel et al., 2013)

This leads to four processes namely domain analysis, requirements analysis, domain implementation and product derivation, each of which is discussed in the following sections with its corresponding realization.

### Domain Analysis

Domain analysis considered requirements engineering for an entire product line and is used to define which features are relevant to be implemented into reusable artefacts, resulting in a feature model. This contains the steps domain scoping and domain modelling. Domain scoping limits the range of desired features that should be supported by the product line. This is done by collecting information about the target domain and making decisions depending on the overall goals the software product line should accomplish. By domain modelling the commonality and variability are captured and documented (Apel et al., 2013, p. 21).

The overall domain scoping consists of two distinct domains that are relevant for the specific case of question generation. The focus is to implement the generator for the use in the learning management system ILIAS, which provides a wide range of features ranging from group and process management tools, access control mechanisms, assessment and exercising capabilities and many other things. Therefore, it is important for the focus of this study to only consider the aspect of assessment, especially focused on the use of single-choice questions, meaning multiple-choice questions with exactly one correct answering alternative. Additionally, the generator shall be able to be used to create questions for computer science and programming assessment. Based on these domains common and mandatory features are derived, that are necessary to create a question in the first place.

By the use of these domains, there are still many features that could be considered for question generation, such as the integration of files, interactive images or a media player. For a feasible prototype with the focus on computer science and programming assessment via single-choice questions the scope covered by the generator needed to be limited. For this reason, the following mandatory and optional features were as sharply defined as possible.

Mandatory features of a question are the name of the author, the name of the question, the question/task itself, at least one correct answer option and at least one false answer option. Since we wanted to develop the generator for practical use at our institute, it was determined that each question must contain exactly one correct and three incorrect answer alternatives. In addition to these parts, there were multiple variable features for a question, which were derived directly from the system or were added by us as the stakeholders.

**Fig. 2:**
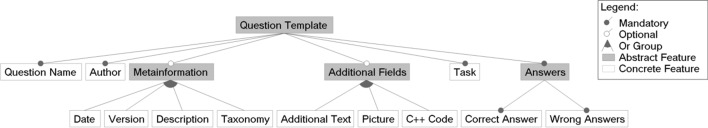
Feature model based on the domain analysis

Important to mention here is, that ILIAS would provide many possibilities to create the questions or parts of questions, but based on the chosen domains, the generator is initially designed to contain only the important features. These optional features consist of additional fields, that may be created per question in ILIAS. These include an additional field of text, a possibility of including a picture and source code. Furthermore, meta-information like date of creation, version or a description of the question may be present, the description can be imported into ILIAS, but the date and version are included to manage the state of the question over multiple iterations. In addition, a question can be part of, or represent a level of a learning taxonomy that can be specified. In total the derived features from the domain analysis are shown in the feature model Fig. 2 based on the presented common notations in Benavides et al. (2010) and Apel et al. (2013). Thereby a concrete feature means that there is a direct implementation artefact, whereas an abstract feature is used for structuring and documentation but is not bound to an implementation artefact.

### Requirements Analysis

In the requirement analysis the specific customer needs get mapped to the identified features from the domain analysis. In the mapping process it is to be determined whether the requirements can be mapped at all and if not, how to proceed. In the case requirements cannot be mapped, it is to be determined if a requirement is out of scope, a next best product can be assembled or if the scope has to be changed and features are to be added (Apel et al., 2013, p. 23).

Based on the developed features in the domain analysis, only precise questions and answers are currently represented and the sole purpose of a generator would be to create import files for such questions. But the goal of the research is to create questions families. This cannot be mapped onto the current features and only partially can this be accomplished by changing the scope of certain features. Therefore, there is a need to develop additional features.

The first change can be made to the available correct and false answers. In making it possible that multiple correct answers and more than three false answers can be presented, a generator would be able to take in the needed amount to create a full question. Secondly with these changes one might want to exclude certain answers based on others that are chosen. This way educators can create similar correct and false answer possibilities that can exclude one another to create a concise question.

To create more variability our approach takes up the basis of generative software development. In turn this means, that new features are created to resemble parameters, their value ranges and potential interactions or dependencies between them. These can be implemented in almost any other content field (e.g. source code, answers, task…) to replace the parameter itself with a value from its value range.

**Fig. 3:**
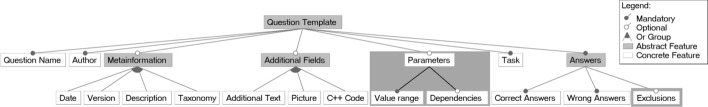
Full feature model for question generation

For the specific question generator, the following Fig. 3 represents all its features based on the domain and requirements analysis. The additional features added through the requirement analysis in comparison to Fig. 2, are highlighted by a darker background.

### Domain Implementation

Inside the process of domain implementation reusable artefacts are to be developed that correspond to the previously identified features. These artefacts usually contain the design, tests, documentation and source code, but this basic idea can also be applied to non-code-artefacts. Since this step specifically targets the solution space, a general implementation strategy has to be chosen to create a reusable framework (Apel et al., 2013, p. 24).

Generally, in product line engineering these strategies would be for example using preprocessor functionality to conditionally include or exclude source code based on variable code or configurations, or to build a framework with the ability to plugin additional features. In translating this to the domain of question generation, each question that in turn relates to a question family has to be separately configured. Therefore, it was decided that every question is to be represented by a separate configuration, which the generator takes as input. Based on the features identified in domain and requirement analysis, the configuration needs to be specified with the mandatory and optional features for each question.

In this way, the generator as a whole is designed to take in any number of configuration files as input for the question generation. These configurations or more precisely templates get parsed by the generator based on the certain criteria for each feature present. These templates are written in a specific format that conforms to the principles of a domain-specific language (DSL). A DSL is a language that is created to solve specific problems in a particular domain (Martin, 2010). The implementation of the DSL expresses itself mainly in form of parameters that can be used throughout the different sections of the question template. Therefore, one could argue that the overall template with its restrictions and characteristics forms a DSL, even if it is only a very limited tiny one. In addition to using the DSL for parameterization, it was decided to extend the DSL to also include markup features like making text appear in bold or italics.

**Fig. 4:**
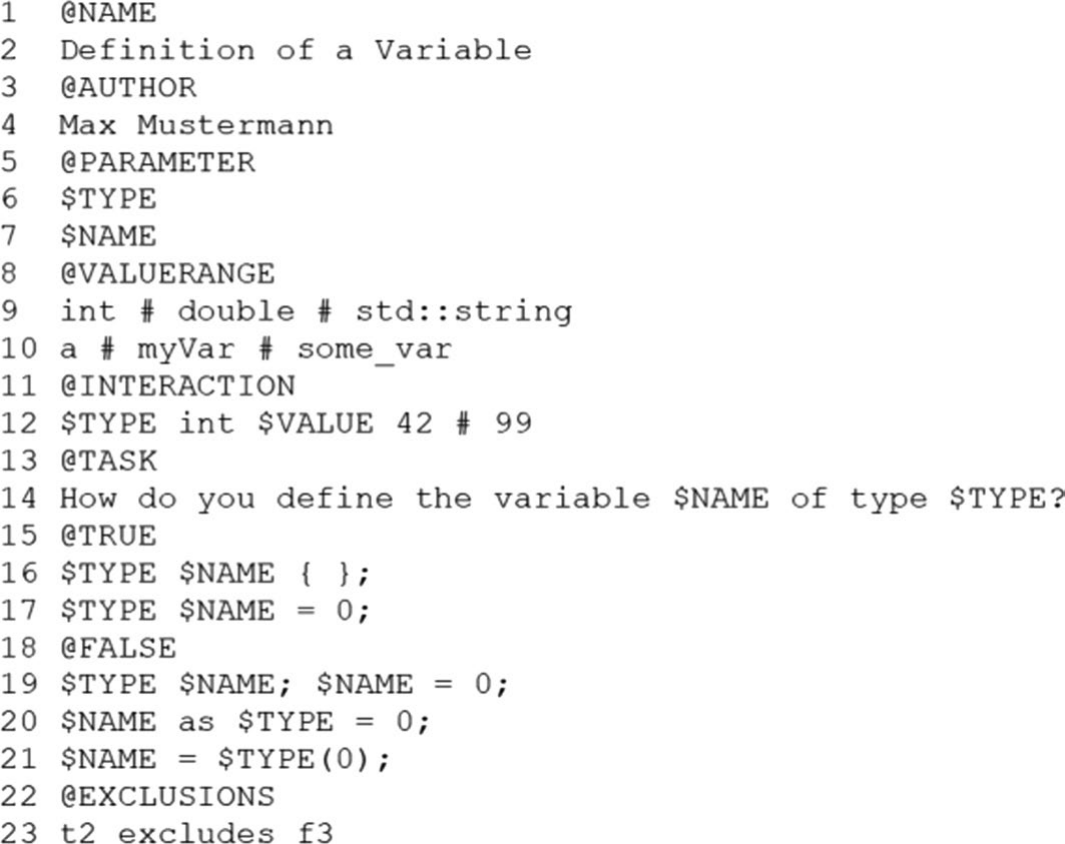
Example use of the domain specific language in the question-template

Based on this, each template consists of at least the five sections that were identified as mandatory features in the domain analysis. In addition, there are the eleven optional sections that can be present. Each section is initiated by the identifier @-character like @AUTHOR and is followed by a predefined structure to efficiently read in files as shown in line 3 and 4 or Fig. 4. Most of the sections simply consist of one or multiple lines that are read by the generator, but especially the sections for parameter, value range, interaction and exclusion do follow some specific structure to allow the user of the generator to parameterize the questions. In the parameter section starting at line 5 in Fig. 4, each line starting with a $-character, creates a one new parameter for the template. Using these parameters like $TYPE in other sections signals the generator that there exists a special functionality, which needs to be separately evaluated. Each parameter then in turn has to be filled with its corresponding values, in the value range section. Again, these are read line by line, so the first specified value range therefore represents the value range of the first specified parameter. The different values are separated by using a #-character. Without using any interactions between parameters, the generator would be able to create all combinations of parameters and their values. To inhibit such behaviour for certain cases of parameters, the author of a template can specify interactions or to say dependencies for certain values. These interactions consist of at least four specified values. The first is the influencing parameter followed by its value, and the third is the influenced parameter followed by at least one of its values, again delimited by the #-character. The last section that is relevant for the question creation is the exclusions. Here the author can specify which answers exclude each other. Since the generator guarantees that a created question has one correct and three item distractors, the exclusions are only necessary if it is important that a true or false answer excludes certain false alternatives, since correct alternatives are never generated together for a question. For the generator, an exclusion is verbalized to consist of an indicator for a true or false answer alternative therefore, “t” or “f” followed by its place in the section. This in turn is followed by “excludes” and then the second indicator which can only be a false answer alternative.

After the template is parsed, the generator proceeds to first generate all valid combinations of answer possibilities and parameters with respect to the set criteria, exclusions or dependencies. When all combinations are created, the questions are put together for either a specified amount, of semi-randomized questions, or all valid combinations of answer possibilities combined with all valid parameter combinations.

For the case that there are no exclusions for answers and no dependencies between parameters the formula for all questions is based on the combination without repetition, multiplied by the number of correct answer possibilities and multiplied by the product of the number of values in the parameter value ranges.

1 $${\text{Formula}}\;{\text{for}}\;{\text{generating}}\;{\text{all}}\;{\text{questions}} = \left\{ {\begin{array}{*{20}l} {{\text{if}}\;p > 0 \Rightarrow c \cdot \left( {\frac{{w!}}{{6 \cdot \left( {w - 3} \right)!}}} \right)} \hfill \\ {{\text{if}}\;p = 0 \Rightarrow c \cdot \left( {\frac{{w!}}{{6 \cdot \left( {w - 3} \right)!}}} \right) \cdot \prod\limits_{{i = 0}}^{n} {p_{i} } } \hfill \\ \end{array} } \right.$$

*c* = number of correct answer possibilities, *w* = number of distractors, *p* = number of parameter values.

As shown in Eq. 1, the formula only holds with the afore-mentioned assumptions, therefore it only represents the maximum number of questions. This maximum number is of course reduced by the exclusions and dependencies. However, the elaboration of this would go beyond the scope of this paper, since the interlocking dependencies and exclusions create a much less predictable number of variants. For example, a simple exclusion that one correct answer excludes a distractor, has a different effect on the number of generatable questions than two distractors that exclude each other, not to speak of the possibility of those interlocking, since an answer possibility might or might not be excluded already by the first exclusion.

**Fig. 5:**
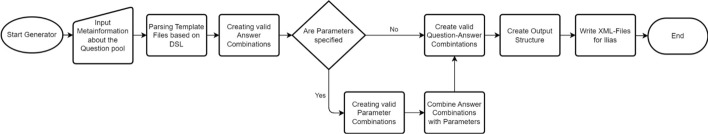
Program flow of the generator

After the generation of all valid questions, the generator proceeds to create a corresponding output for the import into ILIAS. Since the generator is designed to generate whole question pools, a corresponding folder structure and the necessary XML-files are generated. The whole structure can then be easily packed into a Zip-file and imported into ILIAS as a question pool. The programme sequence described here is illustrated again in Fig. 5.

For the development of the generator, a reactive approach was chosen to realize an initial version of the envisioned product line and to progressively further develop the system (Metzger & Pohl, 2014). This way, the generator is developed for the specific use case of computer science single choice questions, but can be extended to meet different needs of other domains. The generator as a whole is written in C++ source code and can simply be compiled and executed using a modern C++ compiler in a command line window.

### Product Derivation

The product derivation or in other cases product—generation, -configuration or -assembly, is the production step of application engineering, where the reusable artefacts are combined with the results of the requirements analysis. This more or less automatable process can involve several other development or customization steps (Apel et al., 2013, p. 25). This in turn results in the product that is to be assembled from the reusable artefacts with additional needs for validating the process and verifying different steps throughout the process.

Overall this step represents the creation of the question-template including the specification of the used parameters. Additionally, a few different steps are checked throughout the generation. This starts with the verification of the parsed template, by using the requirements from the domain and requirements analysis to assess if certain criteria are met, for example, if every parameter has a valid value range. At different stages the validity of the generation gets checked and the question generation is skipped or stopped if it is faulty.

**Fig. 6:**
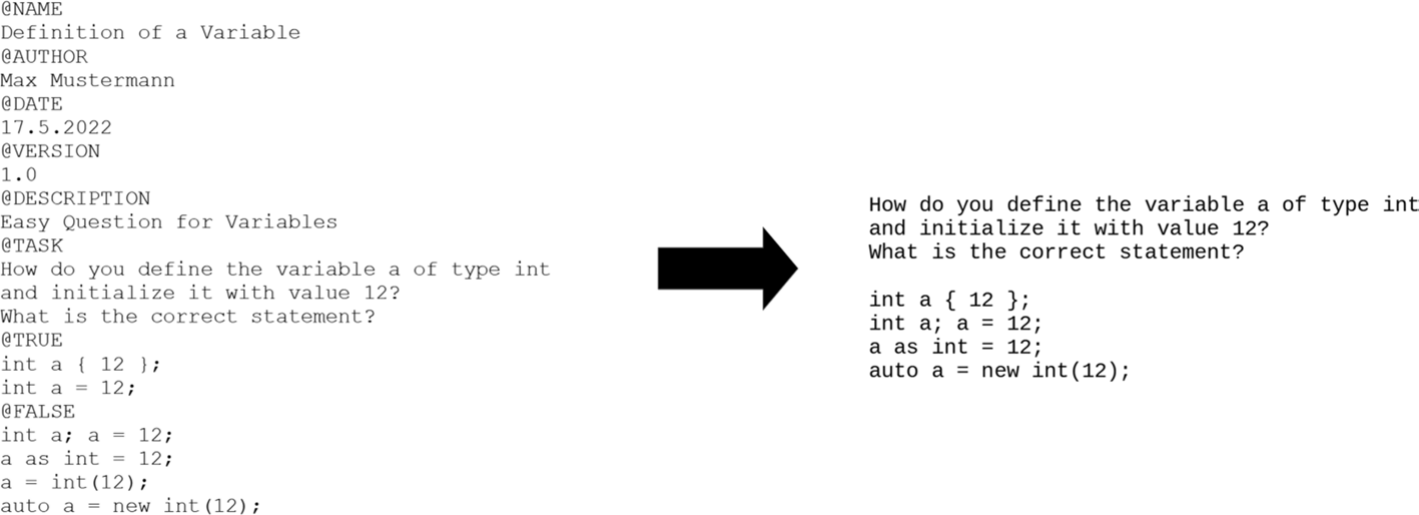
Question template for specialized variable definition and a possible instance

As the creation of the question-template is necessary for the creation of question families, Fig. 6 should present a comprehensible way to use the generator and illustrate how the parameterization can be a valid option for question creation.

Figure 6 shows an example for a question template for the topic how to define and initialize a variable. First, the name of the question, name of the author, date, version number and description of the question are separately defined. After that the actual question, its two true answer possibilities and four distractors are defined. Since there are neither parameters nor exclusions defined, this question template would yield 8 different variants, like one shown on the right.

**Fig. 7:**
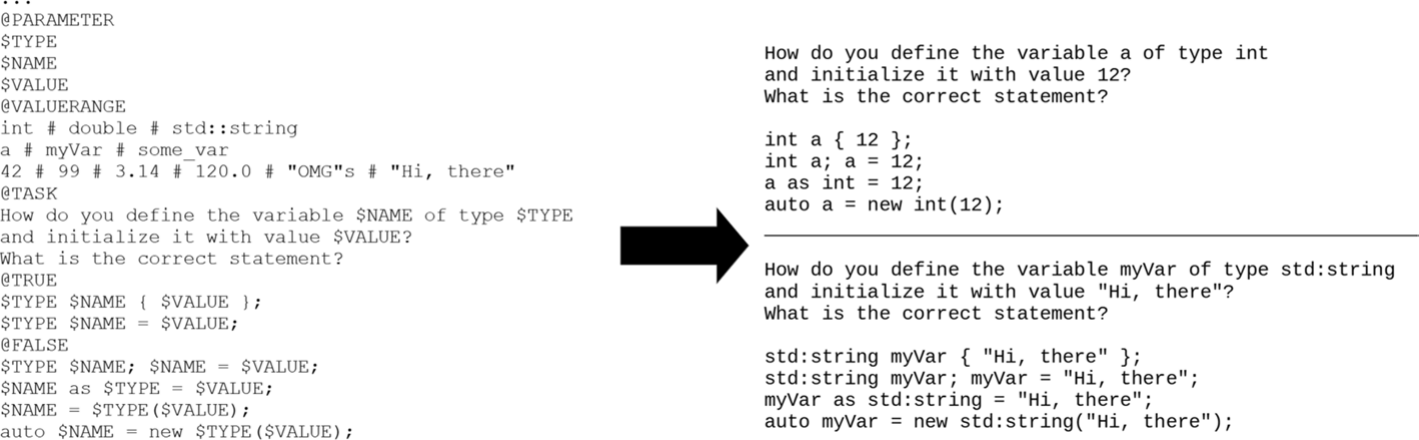
Parameterized question template and example instances

To now express much more variety the question template can be parameterized. As shown in Fig. 7, the parameter and value range sections were added. By introducing a parameter named $TYPE and assigning a value range of the values int, double and std::string to it, the parameter is made available to be used throughout the different template sections. By using the three parameters with a somewhat different number of values, the generator is able to implement these different values into the questions. Therefore, the use of the question-template sections of Fig. 7 as a replacement or extension of the one in Fig. 6 results in 432 possible question.

**Fig. 8:**
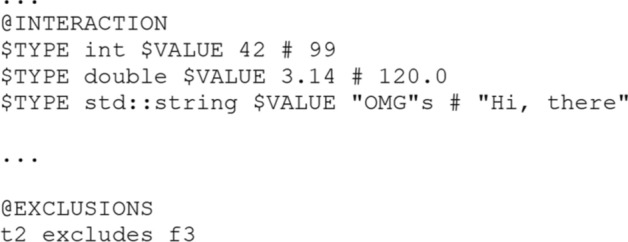
Extensions for eliminating combinations

Of course, the question-template of Fig. 7 is syntactically correct with respect to the generator but semantically the question-template would result in combinations, that would not make sense in the context of programming like having the $TYPE as double and the $VALUE of "OMG"s. Therefore, the extensions of Fig. 8 can be applied to restrict certain question instances. In Fig. 8 for example int is set to the values of 42 and 99, and additionally the second true answer excludes the third false answer. With these restrictions in place the generator would create 90 similar questions based on the 5 valid combinations of correct answers and distractors and the 18 different combinations of parameters.

## Conclusion

As this paper is part of an ongoing research activity, its aim is to introduce a tool and a methodological approach to generate question families based on the principles of software product line engineering, which can be developed further. These principles and corresponding development process were applied to the creation of a single-choice-question-generator. The resulting generator enables educators to design question-templates by the use of a simple and lightweight domain specific language. The templates get generated by either use of multiple manually written answer possibilities, or the use of question and in turn answer parameterization. After the generation process, examiners receive the generated questions as importable files for the use in the learning management system ILIAS.

By the use of this system, a few examinations have been conducted in the summer term of 2022. In result, for example a single-choice exam was conducted with 40 questions for each participant, wherein total 538 different generated questions were used for the examinations. By the use of the generator, the examiners were able to create question-families via the question-templates, so the participants received comparably similar questions, according to the judgement of the examiners. In particular, only one question-template had to be created at a time to generate multiple questions, in turn saving time in creation, quality assurance and import into the system. In addition, the generator will further be used in the following terms for self- and online-assessments so that students can check their own knowledge and understanding.

The generator can easily be used by educators and examiners outside of the computer science field, with or without changes to the question-templates. In addition, it can be adapted for other online learning platforms to use such an approach to semi-automate task generation, where students are provided with similar tasks to improve their skills in the respective field or with specific task types. This would include for example the combination with different learning taxonomies like the revised Bloom taxonomy (Krathwohl, 2002) to create common templates for certain questions that fall into the cognitive learning dimensions and then parameterize them for student exams or exercises.

Finally, such a generator can help educators and examiners when creating questions and students when learning with different iterations of questions. But even though the question-templates in their simple form are easily created, creating complex templates can still be time consuming for complex templates, since the overall question quality depends more on the user and less on the functionality of the generator.

### Limitations and Future Work

Since the questions are not created and parameterized by the generator itself but by the examiner, it is important to highlight that the comparability and difficulty of the answer alternatives is still up to the examiner. Therefore, the actual similarity in difficulty level, could be investigated as the subject of a follow-up study. In addition, by using this kind of question generation for multiple-choice questions the common item analysis methods like Cronbach’s alpha/Tau-equivalent reliability, selectivity and difficulty index are not applicable, since the questions are only similar but otherwise unique with respect to their combination of answer possibilities and parameters. Additionally, most of our exams at the university are conducted with a smaller number of students so that most of the unique questions are only provided to a very small fraction of students, which in turn makes the methods unavailable or very inaccurate. Therefore, it must be explored, which methods can be used to analyze such parameterized tasks, so that in the long term the similarity and difficulty of examinations for different students can be guaranteed. If such methods do not already exist, alternative static quality assurance methods must be developed.

For the generator itself, there are many possibilities to extend its functionality, that can currently be seen as shortcomings. First of all, the used DSL is rather rigid in its applicability. Therefore, certain features can and should be extended or reworked, like the ability to parameterize the picture section to include specific images based on the specified parameters. Additionally, analog to the approach of Nagasaka (2020) it is possible to extend the DSL to be able to specify mathematical formula or to further fill those formula with randomized values.

Although most features are independently defined and implemented, which eases the capability to change them, some features like the parameters, their value range and the dependencies/interactions are strongly coupled, whereby adapting them would require a greater effort. This may also apply to certain features to be changed or newly created, as they have been designed for use in our specific cases, whereby other study courses would have different requirements. As the generator is a research prototype, the source code itself might currently not be a good representation of the principles for software development. Therefore, the generator must be further developed or, if necessary, newly developed, considering various aspects of software engineering. For this reason, additional aspects of software product line management should be introduced, to enable the sharing of artifacts for reuse and adaption.

Additionally, the generator can be extended to generate output for other learning management systems and to generate further question types, surveys or images. For the future work with template-based question generation, the approach is to be further developed to be used for generating programming exercises and images especially various types of diagrams like flowcharts or UML class diagrams.

With the emergence of ChatGPT respectively models of the GPT 03 family and other AI-driven approaches, most online exams can for the most part be nullified. Our approach should be no different, as such a system can provide an answer within seconds and a parameterized task is not a major obstacle, as it would be for a student. Therefore, in the educational domain, research must be done to determine which testing methods can be used to test the skills of students, but which an AI is not capable of. However, it should be noted that the generator is still operational and useful, for example for examinations that are conducted in several rounds, so that students cannot exchange the specific answers. However, through the use of AI-driven approaches like Vimalaksha et al. (2021) and Vachev et al. (2022) future work might want to consider using template-based generators, to generate training data or use the presented mechanisms to create new models.

Finally, it should be pointed out that the system is currently only designed for the parameterization of single choice tasks, which is mainly useful for knowledge testing. As there are many more aspects throughout the learning process, future research should be concerned with investigating to what extent feature orientation of tasks can be used to ensure a better learning process. For example, feature decomposition of tasks can be used to create an adaptive system to help students overcome learning problems, and parameterization can allow students to test their knowledge in a repeatable and independent way.

Generator source code available under: https://github.com/NWillert/Template-basedGeneratorForSingleChoice.

## Footnotes

1 https://featureide.github.io/.

## References

[CR1] Abd Rahim, T. N. T., Abd Aziz, Z., Ab Rauf, R. H., & Shamsudin, N. (2017, November). Automated exam question generator using genetic algorithm. In *2017 IEEE conference on e-learning, e-management and e-services (IC3e)* (pp. 12–17). IEEE. 10.1109/IC3e.2017.8409231

[CR2] Aldabe, I., de Lacalle, M. L., Maritxalar, M., Martinez, E., & Uria, L. (2006). ArikIturri: An automatic question generator based on corpora and NLP techniques. In M. Ikeda, K. D. Ashley, & T. W. Chan (Eds.) *Intelligent tutoring systems. ITS 2006. Lecture notes in computer science* (Vol. 4053). Springer. 10.1007/11774303_58

[CR3] Apel S, Batory D, Kästner C, Saake G (2013). Feature-oriented software product lines.

[CR4] Benavides D, Segura S, Ruiz-Cortés A (2010). Automated analysis of feature models 20 years later: A literature review. Information Systems.

[CR5] Cruz P, Oliveira P, Seabra D (2012). Exercise templates with Sage. Tbilisi Mathematical Journal.

[CR6] Czarnecki, K. (2004). Overview of generative software development. In *International workshop on unconventional programming paradigms* (pp. 326–341). Springer. 10.1007/11527800_25

[CR7] Czarnecki, K., & Eisenecker, U. W. (1999, September). Components and generative programming. In *Software engineering—ESEC/FSE’99* (pp. 2–19). Springer. 10.1007/3-540-48166-4_2

[CR8] Krathwohl DR (2002). A revision of Bloom's taxonomy: An overview. Theory into Practice.

[CR9] Kurdi G, Leo J, Parsia B, Sattler U, Al-Emari S (2020). A systematic review of automatic question generation for educational purposes. International Journal of Artificial Intelligence in Education.

[CR10] Martin F (2010). Domain-specific languages.

[CR11] Metzger A, Pohl K (2014). Software product line engineering and variability management: achievements and challenges. Future of Software Engineering Proceedings.

[CR12] Nagasaka, K. (2020). Multiple-choice questions in mathematics: Automatic generation, revisited. In *The 25th Asian technology conference in mathematics, virtual format, Radford University, Virginia, USA and Suan Sunandha Rajabhat University, Thailand*. https://atcm.mathandtech.org/EP2020/invited/21785.pdf

[CR13] Sewunetie, W. T., & Kovács, L. (2022). Comparison of automatic question generation techniques. In *2022 IEEE 22nd international symposium on computational intelligence and informatics and 8th IEEE international conference on recent achievements in mechatronics, automation, computer science and robotics (CINTI-MACRo)* (pp. 000025–000030). IEEE. 10.1109/CINTI-MACRo57952.2022.10029559

[CR14] Sood SK, Rawat KS (2022). Fog-assisted virtual reality-based learning framework to control panic. Expert Systems.

[CR15] Thüm T, Apel S, Kästner C, Schaefer I, Saake G (2014). A classification and survey of analysis strategies for software product lines. ACM Computing Surveys (CSUR).

[CR16] Vachev, K., Hardalov, M., Karadzhov, G., Georgiev, G., Koychev, I., & Nakov, P. (2022). Leaf: Multiple-choice question generation. In *Advances in information retrieval: 44th European conference on IR research, ECIR 2022, Stavanger, Norway, April 10–14, 2022, Proceedings, Part II* (pp. 321-328). Springer.

[CR17] Vimalaksha, A., Prekash, A., Kumar, V., & Srinivasa, G. (2021, December). DiGen: Distractor generator for multiple choice questions in code comprehension. In *2021 IEEE international conference on engineering, technology & education (TALE)* (pp. 1073–1078). IEEE. 10.1109/TALE52509.2021.9678662

[CR18] Žitko B, Stankov S, Rosić M, Grubišić A (2009). Dynamic test generation over ontology-based knowledge representation in authoring shell. Expert Systems with Applications.

